# Effect of the change of mechanical ventilation mode on cerebral oxygen saturation level in neonates

**DOI:** 10.1186/s12887-023-04036-8

**Published:** 2023-05-10

**Authors:** Jingjing Zhao, Rong Wu, Wei Liu, Manman Li, Wei Wang, Lihua Li

**Affiliations:** 1 Children’s Center, Beijing Luhe Hospital, Capital Medical University, Beijing, China; 2 Yangzhou University Medical College, Neonatal Medical Center, Huai’an Maternity and Child Healthcare Hospital, N.104 South Renmin Road, Huai’an, 223002 China

**Keywords:** Cerebral oxygen saturation, Conventional mechanical ventilation, Mechanical ventilation mode, Neonate, Noninvasive assisted ventilation

## Abstract

**Background:**

This study aimed to apply near-infrared spectroscopy (NIRS) to monitor cerebral oxygen saturation (SrO_2_) level in neonates before and after the change of mechanical ventilation mode, and thus, the effects of the change of mechanical ventilator mode on SrO_2_ level in neonates were assessed.

**Methods:**

This trial was designed as an observational study .A total of 70 neonates who were admitted to the Department of Neonatology of Beijing Luhe Hospital Affiliated to Capital Medical University (Beijing, China) between September 2019 and October 2021 and required respiratory support were included. The variations of SrO2 level before and after the change of mechanical ventilation mode, including changing from Synchronized intermittent mandatory ventilation (SIMV) to noninvasive ventilation (NIV, group 1), and from NIV to oxygen inhalation (group 2), were monitored by Enginmed EGOS-600 A. The changes of SrO_2_ level at 30 min before and 1 h after the change of ventilation mode were compared between the two groups.

**Results:**

The SrO_2_ level in the group 1 30 min before, as well as 10 min, 30 min, and 1 h after the change of ventilation mode was 62.54 ± 3.36%, 65.43 ± 3.98%, 64.38 ± 4.23%, and 64.63 ± 3.71%, respectively. The SrO_2_ level at all the points after the change of ventilation mode increased compared with 30 min before the change (P < 0.05). The SrO_2_ level in the group 2 at each time point was 62.67 ± 4.69%, 64.61 ± 5.00%, 64.04 ± 4.48%, and 64.55 ± 4.32%, respectively. Compared with 30 min before ventilator weaning, the SrO_2_ level at all the points after ventilator weaning increased (P < 0.05). Peak inspiratory pressure (PIP) excluding Nasal Continuous Positive Airway Pressure (NCPAP)) in group 1 was lower than that before extubation, and the difference was statistically significant (P = 0) (Table 7).

**Conclusions:**

SrO_2_ level showed an increasing trend after the change of ventilation mode, and the increase of SrO_2_ level at 10 min after the change of ventilation mode was the most prominent. From SIMV to NIV, increased SrO2 levels may be associated with decreased PIP.

## Background

Mechanical ventilation is an important tool for the treatment of neonatal respiratory failure, which increased neonates’ survival rate [1]. Due to the differences in conditions and devices used in neonatal intensive care unit (NICU), as well as the differences in disease conditions, the mode of mechanical ventilation could be different [2]. The different respiratory supporting modes potentially have different influences on hemodynamics, especially on cardiac output. Studies performed using echocardiography showed that the influences of ventilation modes on hemodynamics were different, while indicators (e.g., cardiac output or blood pressure) only reflect macroscopical circulation, rather than necessarily reflect the oxygenation in blood flow or microcirculation [3–5]. Therefore, although extensive physiological examinations have been performed on neonates undergoing mechanical ventilation, cerebral injury in neonates is still worthy of further investigation. Recently, near-infrared spectroscopy (NIRS) has been applied for noninvasive real-time bedside monitoring of mixed blood oxygen saturation in microcirculation, better reflecting the oxygenation in tissues [6–8]. Therefore, this study utilized the NIRS to investigate the effects of various modes of mechanical ventilation on cerebral oxygen saturation (SrO_2_) level in neonates.The objective of this study was to determine whether the change of ventilator pattern in neonates has any effect on the oxygen supply to the brain tissue. And what is the trend of cerebral oxygen saturation before and after different ventilator mode changes in neonates? The predefined hypothesis was that there was an increase in cerebral oxygen saturation before and after the change of invasive ventilation mode.

## Methods

### Subjects

This trial was designed as an observational study.

Neonates who were admitted to the NICU (with respiratory support) of the Beijing Luhe Hospital Affiliated to Capital Medical University (Beijing, China) between September 2019 and October 2021 were included. The study was approved by the Ethics Committee of the Beijing Luhe Hospital Affiliated to Capital Medical University, and written informed consent was acquired from neonates’ parents 6 h before planned change in ventilator setting. All neonates with respiratory diseases were investigated, in which the respiratory supporting mode changed from one mode to another mode according to the disease conditions.

The inclusion criteria were as follows: (1) age < 28 days; (2) neonates who required respiratory support for respiratory diseases; and (3) signing the informed consent from by neonates’ parents.

The exclusion criteria were as follows: (1) neonates with severe intracranial hemorrhage (grade III or IV); (2) neonates with congenital heart disease or congenital cranial dysinnervation disorders; (3) neonates with severe anemia, severe asphyxia, inherited metabolic diseases, or ischemic or hypoxic cerebral diseases; (4) neonates with incomplete data that could not be used for statistical analysis; and (5) abandoning treatment by neonates’ parents and asking for ventilator weaning.

### Material

The Enginmed EGOS-600 A was used to monitor the SrO_2_ level from 30 min before the change of ventilation mode until 1 h after the change. Spatially resolved NIRS was used in this trial.

**Fig. 1:**
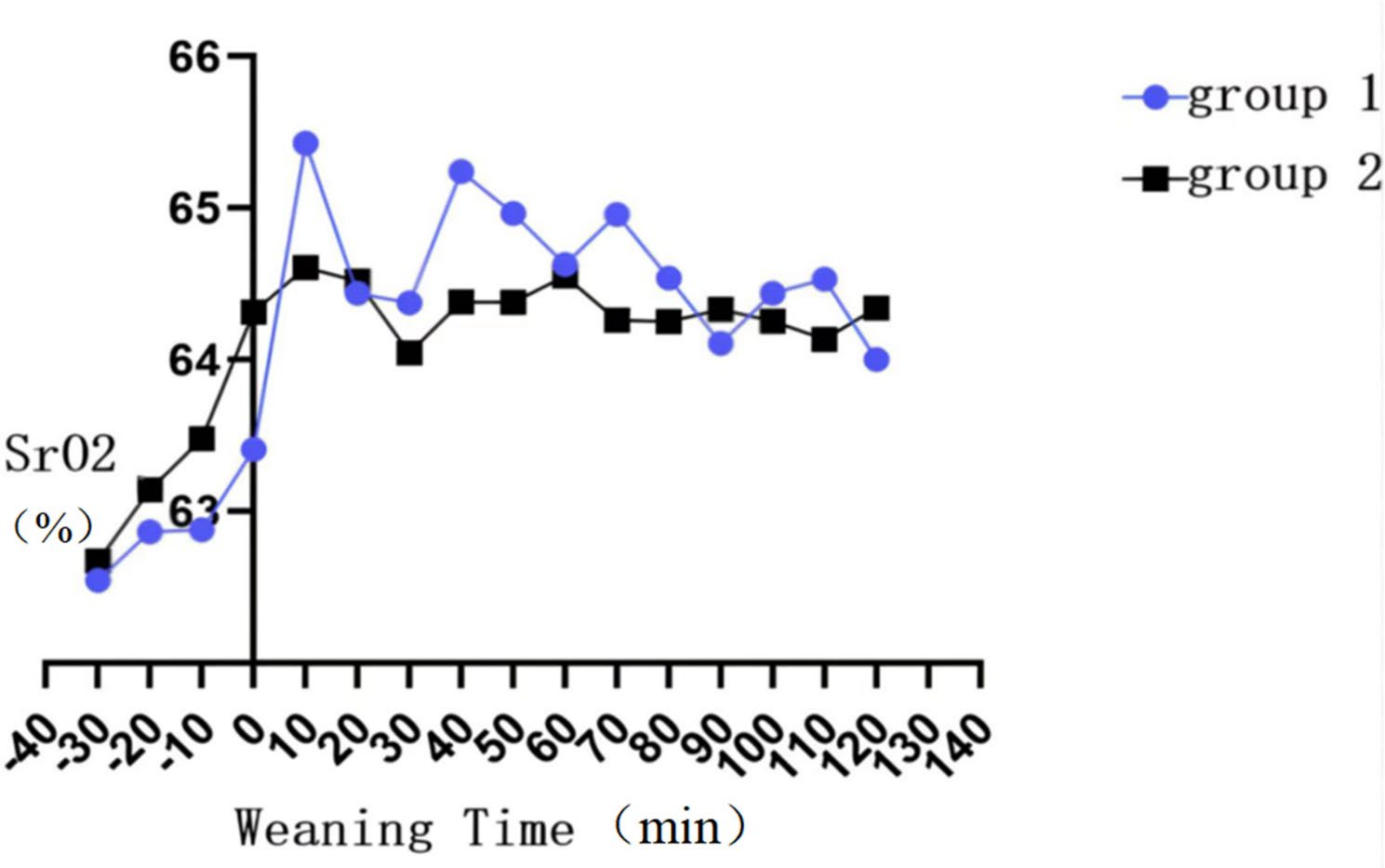
Tendency of changes of SrO_2_ level, respiration, and heart rate with time

The step down in the ventilation strategy from SIMV to non-invasive assisted ventilation including NCPAP/NIPPV/Bilevel positive airway pressure (BiPAP) was denoted as group 1”, and the Noninvasive assisted ventilation (including NCPAP/NIPPV/BiPAP)to oxygen inhalation were denoted as group 2.In addition, the multifunctional electrocardiogram (ECG) monitoring was applied to concurrently monitor clinical indicators, including heart rate, blood pressure, and respiration rate. We then recorded NIRS data for the 30 min before ventilator change and 60 min after ventilator change,then we compared SrO2 data for a 30-minute epoch prior to the change (eg-30 to 0 min, where ventilator change was designated time 0 ) with a 60-minute epoch after the change (e.g. 0 to + 60 min). NIRS saturation data were averaged within 1-minute (NIRS records data every 2 s).The average of SrO_2_ level was calculated every 10 min, and the time-SrO_2_ tendency chart was plotted (Fig. 1). The trend of SrO_2_ level was evaluated. In addition, the time point with a relatively substantial change of SrO_2_ level was selected for statistical analysis. Besides, the influences of gestational age, gender, delivery mode, birth weight, hemoglobin (Hb) level, 1-min Apgar score, and blood-gas indicators (PH, PCO_2_, PO_2_, HCO_3_, SO_2_, and Hb) on variation of SrO_2_ level were investigated. Furthermore, the associations of heart rate, respiration, and blood pressure with variation of SrO_2_ level were explored.

### Statistical analysis

Normally distributed quantitative data were described as mean and standard deviation. The comparisons of SrO_2_ level at different time points within group were performed by the analysis of variance (ANOVA) for repeated measurements, and the Fisher’s least significant difference (LSD) test was used for making comparison between the two groups. The variation of SrO_2_ level in neonates at 10 min after ventilator weaning was used as the dependent variable, and gestational age, birth weight, Hb level, 1-min Apgar score, and blood-gas indicators (PH, PCO_2_, PO_2_, HCO_3_, SO_2_, and Hb) were used as the independent variables, and then, the multivariate logistic regression model was established.The paired T-test was used to compare the changes of parameters before and after the change of ventilator mode. The vital signs at different time points were compared by ANOVA for repeated measurements. P < 0.05 was considered statistically significant.

## Results

### General characteristics

Totally, 75 neonates met the inclusion criteria, of whom 5 neonates were excluded. Specifically, 1 neonate with ischemic or hypoxic cerebral disease was excluded, 2 neonates were excluded for the incomplete data of SrO_2_ monitoring, and 2 neonates with congenital heart disease were excluded. Therefore, 70 neonates were finally included in this study, in which the gestational age ranged from 26 + 2 to 40 + 2 weeks, and the mean body weight was 2122.5 ± 800.2 g (Table 1).

**Table 1:** Clinical characteristics of the included neonates

Group	Gestational age (w,‾x ± s)	Weight (g, ‾x ± s)	Age (d) of ventilator weaning		Gender		Delivery mode		Hb	
			< 72 h (n)	> 72 h (n)	Male (n)	Female (n)	Spontaneous delivery (n)	Caesarean section (n)	Normal (n)	Anemia (n)
Total (n = 70)	33.1 ± 3.3	2122.5 ± 800.2	37 (53%)	33 (47%)	45 (64%)	25 (36%)	12 (17%)	58 (83%)	34 (48%)	36 (52%)
Group 1	32.3 ± 3.5	1862.0 ± 801.1	18 (48%)	19 (57%)	26 (71%)	11 (29%)	5 (14%)	32 (86%)	15 (40%)	22 (60%)
Group 2	34.4 ± 2.3	2399.1 ± 638.7	19 (52%)	14 (43%)	19 (58%)	14 (42%)	7 (21%)	26 (79%)	19 (57%)	14 (43%)

### Influences of clinical characteristics on SrO_2_ level

The multivariate logistic regression analysis showed that birth weight, gestational age, 1-min Apgar score, Hb level, blood-gas indicators (PH, PCO_2_, PO_2_, HCO_3_, SO_2_, and Hb), age (d) of ventilator weaning, gender, and delivery mode had no significant influence on variation of SrO_2_ level (P > 0.05) (Table 2).

**Table 2:** Multivariate logistic regression analysis of the influences of clinical characteristics on SrO_2_ level

Model			*B*	Standard error	Trial	*t*	*P*
	Birth weight		0	0.001	0.146	0.571	0.57
	Gestational age		-0.046	0.206	-0.059	-0.222	0.825
	Apgar score (1-min)		0.11	0.163	0.084	0.672	0.505
	Hb		0.014	0.016	0.109	0.877	0.384
Blood-gas indicator changes	PH		-1.045	6.56	-0.029	-0.159	0.874
	PCO2		-0.036	0.055	-0.111	-0.664	0.509
	PO2		0.014	0.013	0.16	1.134	0.262
	cHCO3		-0.292	0.234	-0.18	-1.247	0.217
	ctHb		-0.019	0.017	-0.124	-1.083	0.284

Blood-gas indicator changes refer to the changes of blood-gas indicators before and after the change of mechanical ventilation mode. * P ≤ 0.05 indicated the presence of statistical significance

Correlation analysis showed that with the change of mechanical ventilation mode, heart rate, respiration rate, and SrO_2_ level also changed concurrently (Fig. 1). Blood pressure monitoring showed that both systolic blood pressure (SBP) and diastolic blood pressure (DBP) increased after than before ventilator weaning. Therefore, with the change of ventilation mode, the heart rate, respiration rate, and blood pressure all slightly increased concurrently with the SrO_2_ level; however, ANOVA for repeated measurements showed that the changes were not statistically significant (P > 0.05) (Table 3).

**Table 3:** ANOVA of heart rate, respiration and transcutaneous oxygen saturation before and after mechanical ventilation mode change

	30 min before ventilator weaning	10 min after ventilator weaning	30 min after ventilator weaning	60 min after ventilator weaning	*F*	*P*
Respiration	41.3 ± 5.3	42.3 ± 5.4	41.6 ± 6.5	41.3 ± 3.6	0.587	0.672
Heart rate	136.4 ± 10.3	137.9 ± 10.8	138.4 ± 10.4	136.7 ± 5.4	2.333	0.065
Percutaneous SO_2_	94.8 ± 2.3	94.6 ± 2.0	94.6 ± 2.1	94.6 ± 2.2	1.581	0.181
SBP	69.78 ± 6.40	70.42 ± 5.49	70.60 ± 6.30	--	0.349	0.706
DBP	37.36 ± 5.40	37.61 ± 4.91	38.37 ± 5.25	--	0.719	0.488

* P ≤ 0.05 indicated the presence of statistical significance

### *Influences of changing from* SIMV *to noninvasive ventilation (NIV) mode on SrO*_***2***_ *level*

**Fig. 2:**
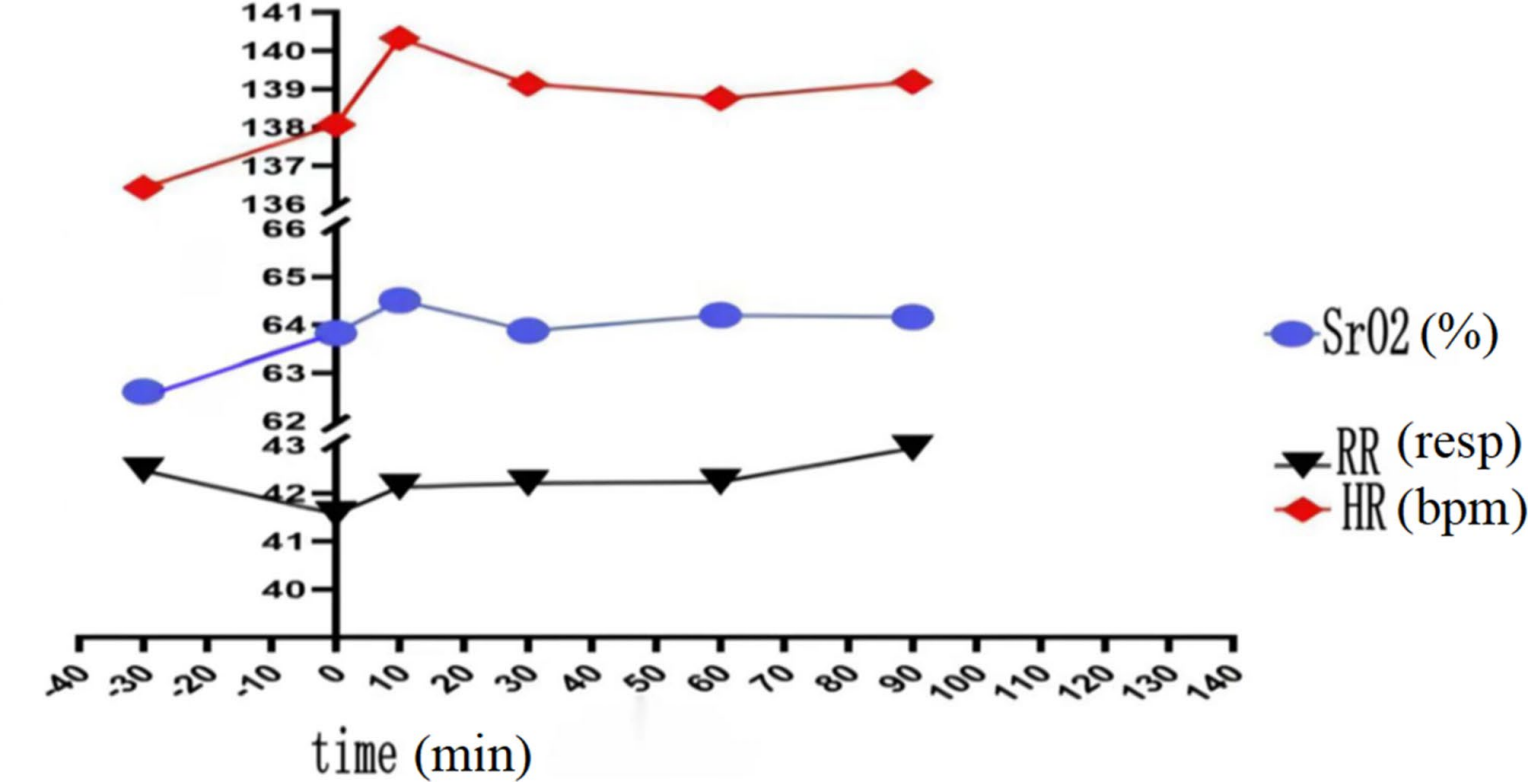
Tendency of the impact of the change of mechanical ventilation mode on SrO_2_ level. SrO_2_ level tended to increase after the change of mechanical ventilation mode in the group 1 and group 2, which reached the peak at 10 min after the change. The variation of SrO_2_ level in the group 1 was more prominent, and the change was larger

A chart was plotted to illustrate the tendency of change of SrO_2_ level with time in the group 1, which showed that the SrO_2_ level tended to increase after ventilator weaning, reached the peak at 10 min after ventilator weaning, and then started to decrease, while the SrO_2_ level was still higher than that before ventilator weaning (Fig. 2). The linear analysis of the change of SrO_2_ level with time in the group 1 showed that the change had a linear tendency (*F* = 10.796, *P* = 0.002) (Table 4). The SrO_2_ level in neonates before ventilator weaning was 62.54 ± 3.36% in the group 1, and the ANOVA for repeated measurements showed that the SrO_2_ levels at the 4 time points were statistically significant (P < 0.001). The SrO_2_ level at 10 min after ventilator weaning (65.43 ± 3.98%) was significantly higher than that before ventilator weaning (P < 0.001); however, the SrO_2_ level at 30 min (P < 0.001) and 1 h (F = 10.524, P < 0.001) after ventilator weaning decreased gradually (Fig. 2).

**Table 4:** Linear analysis of the change of SrO_2_ level with time

	Time	*F*	*P*
Group 1	Linear	10.796	0.002^*^
Group 2	Linear	7.717	0.009^*^

The SrO_2_ level in neonates with the change from invasive to noninvasive respiratory support showed a linear tendency with the change of time (P = 0.002). The SrO_2_ level in neonates with the change from noninvasive respiratory support to oxygen inhalation also showed a linear tendency with the change of time (P = 0.009)

### Influences of changing from NIV to oxygen inhalation on SrO_2_ level

A chart was plotted to illustrate the tendency of the change of SrO_2_ level with time in the group 2, which showed that the SrO_2_ level also tended to increase after ventilator weaning. The SrO_2_ level reached the peak at 10 min after ventilator weaning and then started to decrease, while the SrO_2_ level was still higher than that before ventilator weaning (Fig. 2). The linear analysis of the change of SrO_2_ level with time in the group 2 also showed that the change had a linear tendency (*F* = 7.717, *P* = 0.009) (Table 5). The SrO_2_ level in neonates before ventilator weaning was approximately 62.67 ± 4.69% in the group 2, which significantly increased to 64.61 ± 5.00% at 10 min after ventilator weaning (P = 0.002), and then, started to decrease gradually 10 min later and stabilized at 30 min after ventilator weaning. However, the SrO_2_ level (64.55 ± 4.32%) was still significantly higher than that before the ventilator weaning (P = 0.003) (Table 6).

**Table 5:** ANOVA for repeated measurements of SrO_2_ level in 37 neonates in group 1

Time	$\bar {X} \pm s$	*F*	*P*
30 min before mode change	62.54 ± 3.36	10.524	< 0.001^*^
10 min after mode change	65.43 ± 3.98		
30 min after mode change	64.38 ± 4.23		
1 h after mode change	64.63 ± 3.71		

The SrO_2_ level between 30 min before and 10 min after mode change was significantly different (P < 0.001); the SrO_2_ level between 30 min before and 30 min after mode change was significantly different (P = 0.001); the SrO_2_ level between 30 min before and 1 h after mode change was significantly different (P < 0.001); the SrO_2_ level between 10 and 30 min after mode change was significantly different (P = 0.037)

**Table 6:** ANOVA for repeated measurements of SrO_2_ level in 33 neonates in group 2

Time	$\bar {X} \pm s$	*F*	*P*
30 min before ventilator weaning	62.67 ± 4.69	4.062	0.016^*^
10 min after ventilator weaning	64.61 ± 5.00		
30 min after ventilator weaning	64.04 ± 4.48		
60 min after ventilator weaning	64.55 ± 4.32		

The SrO_2_ level between 30 min before and 10 min after ventilator weaning was significantly different (P = 0.002); the SrO_2_ level between 30 min before and 30 min after ventilator weaning was significantly different (P = 0.011); the SrO_2_ level between 30 min before and 1 h after ventilator weaning was significantly different (P = 0.003)

### Comparison of ventilator parameters

PEEP in group 1 was lower than that before extubation, and FiO2 was higher than that before extubation, but the differences were not statistically significant (P = 0.34,P = 0.156). However, PIP (excluding NCPAP) in group 1 was lower than that before extubation, and the difference was statistically significant (P = 0) (Table 7). FiO2 in group 2 was higher than that before ventilator weaning,but the differences were not statistically significant (P = 0.148) (Table 7).

**Table 7:** T-test of ventilator parameters

	group1				group2
	PEEP	FiO2	PIP		FiO2
before change	5.16 ± 0.37	27.91 ± 4.66	18.25 ± 0.85		27.30 ± 7.84
after change	5.09 ± 0.19	28.97 ± 3.98	16.51 ± 1.65		29.27 ± 1.39
t	1.044	-1.45	6.096		-1.482
*p*	0.304	0.156	0*		0.148

* P ≤ 0.05 indicated the presence of statistical significance

## Discussion

The findings of this study demonstrated SrO_2_ level increased in both groups after the change of mechanical ventilation mode.Birth weight, gestational age, 1-min Apgar score, Hb level, and blood-gas indicators had no significant influence on neonatal SrO_2_ level. From SIMV to NIV, increased SrO2 levels may be associated with decreased PIP.

### Influences of changing from SIMV to NIV on SrO_2_ level

The findings of the present study showed that after the change of mechanical ventilation mode, including from SIMV to NIV, neonatal SrO_2_ level increased compared with before the change, which reached the peak at 10 min after the change, and then gradually stabilized at 1 h after the change.

Schwaberger et al. have shown an influence of respiratory support on cerebral hemodynamics (cerebral blood volume, cerebral oxygenation) [9, 10]. The effect of invasive ventilation on SrO_2_ could be associated with the increase of intrathoracic pressure caused by the ventilation, which induced cerebral venous stasis and cardiac venous return impairment, and could consequently influence the cerebral blood volume [11]. In addition, invasive procedures changed the anatomical structures of the respiratory tract; the neonate-machine interaction could also induce the variation of arterial blood pressure and cerebral blood flow velocity, and could consequently lead to the variation of SrO_2_ level [12, 13]. Govindan et al. continuously monitored the mean arterial pressure (MAP) and cerebral blood flow in neonates who underwent positive pressure ventilation by NIRS, and found that the variation of ventilator-related cerebral blood volume in neonates could further induce the passive change of cerebral oxygen metabolism, and consequently induce cerebral injuries in neonates [14]. Several studies have recently compared the effects of different invasive ventilation modes on cerebral oxygen. In a study performed by Mrozek et al. [15], intermittent mandatory ventilation, synchronized intermittent mandatory ventilation, and assist/control (A/C) mode of mechanical ventilation were used for neonates with respiratory stress syndrome within 6 h after the application of a surfactant, and the findings showed that neonate-machine interaction could induce arterial pressure variation, and further induce cerebral blood flow instability, thereby inducing severe intraventricular hemorrhage. Schwaberger B et al. found that the oxygen supply for brain increased within 15 min after birth in healthy neonates, which induced the contraction of cerebral blood vessels and reduced cerebral blood volume [9, 16]. The present study compared neonates who underwent mechanical ventilation and those who did not undergo mechanical ventilation, and found that the blood volume in both groups decreased continuously within 15 min after birth, while the amplitude of reduction in neonates who underwent mechanical ventilation was relatively low within the first 7 min [9]. However, there are still debates on whether the reduction of cerebral blood volume can be induced by mechanical ventilation or the pathological states in neonates.

### Influences of changing from NIV to oxygen inhalation on SrO_2_ level

The findings showed that in the noninvasive-oxygen inhalation group (group 2), neonatal SrO_2_ level increased compared with before ventilator weaning, which reached the peak at 10 min after the ventilator weaning and then gradually stabilized.

Theoretically, the influence of changing from NIV to oxygen inhalation on SrO_2_ level could be associated with the disappear of pressure from assisted ventilation, which induced the reduction of intrathoracic positive pressure, increased venous return and cardiac output, and consequently increased the cerebral blood flow and induced the elevation of SrO_2_ level [17, 18]. However, Schwaberger et al. have shown that respiratory support immediately after birth by using sustained lung inflations in preterm infants did not show significant differences in CBV and cTOI compared to not using RS [19].Zhong et al. [4]investigated 26 neonates (born at the gestational age of 25–37 weeks), and found that the mean cerebral oxygenation in neonates on positive airway pressure ventilation was not significantly different from that in the normal control group; however, mean cerebral fractional oxygen extraction (FOE) in neonates increased from 0.22 ± 0.10 to 0.28 ± 0.13 (*P* = 0.002) with the increase of time of positive airway pressure ventilation, indicating the increasing tendency of SrO_2_ level.

## Conclusions

In summary, SrO_2_ level increased in neonates after changing from SIMV to NIV mode, which reached the peak at 10 min after the change of mode and then gradually stabilized at 30 min after the change. The SrO_2_ level also increased in neonates after changing from NIV to oxygen inhalation, which peaked 10 min after the change and then gradually stabilized at 10 min after the change. The change of SrO_2_ level from SIMV to NIV may be associated with decreased PIP. .

## Data Availability

All data generated or analysed during this study are included in this article.

## List of Abbreviations

NIRS: Near-infrared spectroscopy
SrO2: Cerebral oxygen saturation
CMV: Conventional mechanical ventilation
SIMV: Synchronized intermittent mandatory ventilation
NIPPV: Nasal intermittent positive pressure ventilation
BiPAP: Bilevel mask positive airway pressure
NCPAP: Nasal continuous positive airway pressure
NICU: Neonatal intensive care unit
ECG: Electrocardiogram
Hb: Hemoglobin
ANOVA: Analysis of variance
LSD: Least significant difference
SBP: Systolic blood pressure
DBP: Diastolic blood pressure
